# A comparative analysis of high-flux and low-flux dialysis in cervical cancer patients with obstructive renal failure showing no significantly improved renal function after catheterisation

**DOI:** 10.12669/pjms.37.4.3515

**Published:** 2021

**Authors:** Chen-li Zhang, De-qiong Xie, Li-na Ao, Lei Zhu

**Affiliations:** 1 Chen-li Zhang, Department of Nephrology, The Second People’s Hospital of Yibin City, Yibin, Sichuan, 644000, P.R. China; 2 De-qiong Xie, Department of Nephrology, The Second People’s Hospital of Yibin City, Yibin, Sichuan, 644000, P.R. China; 3 Li-na Ao, Department of Nephrology, The Second People’s Hospital of Yibin City, Yibin, Sichuan, 644000, P.R. China; 4 Lei Zhu, Department of Nephrology, The Second People’s Hospital of Yibin City, Yibin, Sichuan, 644000, P.R. China

**Keywords:** KEYWORDS: Cervical cancer, High-flux dialysis, Obstructive nephropathy, Treatment

## Abstract

**Objective:**

This study aims to compare the clinical application value of high-flux dialysis with low-flux dialysis in patients without significantly improved renal function after cervical cancer and obstructive renal failure catheterisation.

**Methods:**

This prospective randomised study was conducted from January 2018 to December 2019. Eighty cervical cancer patients with obstructive renal failure who showed no significant renal function improvement after catheterisation were randomised into two groups (n = 40 in each group) in the Second People’s Hospital of Yibin City. High-flux and low-flux dialysis were employed in the experimental group and the control group, respectively. Treatments in both groups were provided every other day, with the whole course lasting one week. Data were recorded before and after dialysis included inflammatory factors such as IL-6, CRP and TNF-a, large and moderate molecular toxins (e.g., β2 micro-globulin, parathyrin (PTH) and cysteine protease inhibitor). Renal function changes during the dialysis were also recorded. Afterwards, the two groups were compared regarding the overall efficacy.

**Results:**

Both the experimental group and the control group experienced a significant decrease in IL-6, CRP, TNF-a, β2 micro-globulin, PTH and cysteine protease inhibitor, with the decrease in the experimental group being more evident (p < 0.05). After dialysis was completed, the experimental group restored renal function indicators such as Cre, CysC and serum K+ levels more quickly than the control group (p < 0.05). The effective rate was 100% for the experimental group and 87.5% for the control group. The intragroup difference in the efficacy.was significant.

**Conclusions:**

High-flux dialysis appears to be more beneficial for cervical cancer patients with obstructive renal failure, showing no significant improvement in renal function after catheterisation. It restored renal function more quickly, had more radical draining of inflammatory factors and large and moderate molecular toxins, and had a higher overall effective rate.

## INTRODUCTION

Cervical cancer is a common gynaecological cancer with high morbidity. It is the fourth most common malignant tumour among women worldwide, and its morbidity is much higher in developing countries than in the developed ones.^1^ For advanced cervical cancer, renal function will be inevitably impaired with hydronephrosis caused by all sorts of factors^2^, including extensive hysterectomy, pelvic lymphadenectomy, damage of pelvic chemoradiotherapy to the ureter, and direct invasion of the tumour to the ureter. Renal function impairment is usually postrenal and obstructive. Unlike primary renal diseases, it usually features acute onset and fast progression and may lead to renal failure, electrolyte disturbance and toxicant build-up within a short period. Though immensely harmful, renal function impairment can quickly recover once the obstruction is relieved by such surgical intervention measures as ureter catheterisation.^3^ However, a small portion of patients may appear to have a slight improvement in post-surgical renal function so that they may still need further haemodialysis to improve the renal function and electrolyte disturbance, correct the state of internal toxicant accumulation, and get steadily through the critical stage to fight for a better chance for subsequent treatment.

This study aimed to further illustrate the clinical application value of high-flux dialysis by comparing the clinical effects of high-flux and low-flux dialysis in dealing with cervical cancer patients that have insignificantly improved renal function after obstructive renal failure catheterisation.

## METHODS

This is a prospective, randomised controlled study. From January 2018 to December 2019, 80 patients were enrolled and then randomised into two groups (n=40 in each group) in the Second People’s Hospital of Yibin City. Patients in the experimental group aged between 46 and 67 years old with an average age of 56.23 ± 6.84 years. It required from 1.3 to 3 years to develop from cervical cancer to obstructive renal failure (average: 2.32 ± 0.49 years). By contrast, control group individuals aged between 44 and 66 years old with an average age of 56.00 ± 8.14 years. The disease course lasted between 1.4 and 2.7 years (average: 2.33 ± 0.36 years). The general data of both groups of the patient (Table-I) were comparable and did not differ significantly (*p* > 0.05).

**Table-I:** A comparison of patients’ general data in the two groups (*X̅* ±S), n = 40.

Indicator	Experimental	Control	t/χ^2^	*P*
Age (year)	56.23 ± 6.84	56.00 ± 8.14	0.14	0.89
Course (year)	2.32 ± 0.49	2.33 ± 0.36	0.10	0.92
Cause				
Direct invasion (no, %)	13(32.5%)	15(37.5%)	0.22	0.17
Pelvic chemoradiotherapy (no, %)	18(45%)	18(45%)	0.00	0.18
Pelvic surgery (no, %)	9 (22.5)	7(17.5)	0.31	0.19

***Note:*** *p* >0.05.

### Ethical approval

The study was approved by the Institutional Ethics Committee of the Second People’s Hospital on August 15, 2020, of Yibin City, and written informed consent was obtained from all participants

### Inclusion criteria:

1. Patients with advanced cervical cancer, combined with obstructive renal failure, showed no significant improvement in renal function after ureteral catheterisation (serum creatinine >707 μmol/L) and serum potassium >6.5 mmol/L and compliance with haemodialysis criteria.^4^
2. Patients who were compliant and could complete the study.
3. Patients who agreed to join the study and provided informed consent.

### Exclusion criteria:

1. Patients with significantly improved renal function after catheterisation (serum creatinine >600 μmol/L; serum potassium>6.0 mmol/L) did not need dialysis.
2. Severely consumed patients with bad general conditions.
3. Patients with other mental diseases, imperfect compliance or inability to participate in the study until it ended.
4. Patients turning to other therapies due to improperly indwelling catheter or unsuccessful catheterisation.

### Treatment Catheterisation Methods

The patient was asked to lie in the lithotomy position to accept topical anaesthesia, sterilisation and surgical draping before the transurethral ureteroscope was inserted into the bladder. Once the ureteral orifice was located, a Zebra urological guidewire was put into the ureter to guide the stent into the ureter. When the stent was adjusted to a satisfactory position, the other stent was indwelled adequately in the same way. An abdominal X-ray was performed immediately after the operation was done to determine the locations of the stents. Stents were replaced every six months afterwards.

### Types of Haemodialysis Methods - Experimental group

High-flux dialysis was chosen. A German Braun 710207t haemodialysis machine was used to perform the dialysis with bicarbonate solution as the dialysate. The dialyzer model was an FX80 polysulfone membrane with an ultrafiltration coefficient of 46 ml/(mm Hg h), a membrane area of 1.8 m^2^. The dialysis was done every other day for four hour each time.

### Control group

Low-flux haemodialysis was selected with an FX8 polysulfone dialyzer. The ultrafiltration coefficient was 12 ml/(mm Hg h) (1 mm Hg = 0.1333 kPa), and the membrane area was 1.0 m^2^. The dialysis was done every other day for four hour each time. Both groups received one-week dialytic treatment.

Indicators of blood microinflammation and renal function: Fasting blood in the morning was collected from all patients before dialysis. One week after dialysis to examine changes in microinflammation reaction, large and moderate molecular toxins and renal function. The microinflammation reaction evaluation included IL-6, CRP and TNF-α. As for large- and moderate-molecular toxins, such indicators as β2 micro-globulin, parathyrin (PTH) and cysteine protease inhibitor were examined to assess their changes before and after dialysis. For renal function assessment, morning blood was sampled before dialysis, and every day after dialysis, it was tested with a fully automatic biochemical analyser. The analysis covered creatinine, urea nitrogen and cystatin. The time required for renal function indicators to recover was recorded, and the intragroup difference in this respect was compared.

### Efficacy assessment indicators:

**Excellent:** Both BUN and Cr significantly improved after treatment, and almost all clinical symptoms disappeared.

**Effective:** After treatment, BUN and Cr improved somewhat, and the clinical symptoms were alleviated.

**Ineffective:** No significant improvement was witnessed in the clinical symptoms; BUN and SCr revealed no evident changes. The effective rate = (excellent + effective)/total cases × 100%. The time for treatment and renal function improvement was recorded.

### Statistical analysis:

All data were processed using SPSS 20.0. Metrological data were presented as ± S. Data analysis between the experimental and the control groups was checked with an independent-samples t-test, whereas the rate comparison was checked with χ^2^. *P* < .05 was considered statistically significant.

## RESULTS

### Indicators of microinflammation reaction

Both the experimental group and the control group displayed a significant decrease in indicators, such as IL-6, CRP and TNF-α after treatment was completed (*p* = .00). However, the experimental group’s decrease appeared to be more significant than in the control group (IL-6, CRP: *p* = .01; TNF-a, *p* = 0.02) (Table-II).

**Table-II:** A comparison of inflammatory factors in the experimental and control groups before and after treatment (*X̅* ± S), n = 40.

Observed indicator	IL-6(ng/L)				CRP (mg/L)				TNF-α(ng/L)			

Group	Before*	AfterΔ	t	*p*	Before*	AfterΔ	t	*p*	Before*	AfterΔ	t	*p*
ExperimentalΔ	15.43 ± 5.32	8.97 ± 2.24	7.08	0.00	3.37 ± 0.15	2.01 ± 0.47	17.43	0.00	47.52 ± 13.81	20.37 ± 7.24	11.01	0.00
ControlΔ	15.33 ± 4.68	10.23 ± 1.72	6.47	0.00	3.41 ± 0.82	2.25 ± 0.33	8.30	0.00	46.73 ± 12.55	23.72 ± 5.48	10.63	0.00
t	0.09	2.82			0.30	2.64			0.27	2.33		
*p*	0.93	0.01			0.76	0.01			0.79	0.02		

***Note:*** before = before dialysis but after catheterisation; after = 1 week after the dialysis,

* *p* > .05, Δ*p* < 05.

### Indicators of changes in large- and moderate-molecular toxins

In both the experimental group and the control group, β2 micro-globulin, PTH and cysteine protease inhibitor presented a significant decrease after the treatment. The differences were significant (*p* = 0.00), with those in the experimental group being more striking. The intragroup differences were also significant (β2 micro-globulin: *p* = 0.03; PTH: *p* = 0.00; and cysteine protease: *p* = 0.01) (Table-III).

**Table-III:** An intragroup comparison of large and moderate molecular toxins before and after the treatment (*X̅* ± S), n = 40

Observed indicator	β_2_ micro-globulin (mg/L)				PTH (pg/ml)				Cysteine protease inhibitor (mmol/L)			

Group	Before*	After Δ	t	*p*	Before*	AfterΔ	t	*p*	Before*	AfterΔ	t	p
Experimental Δ	19.23 ± 5.02	9.07 ± 3.24	10.75	0.00	667.20 ± 34.47	431.21 ± 23.78	35.64	0.00	5.52 ± 1.24	2.13 ± 0.72	14.95	0.00
Control Δ	19.41 ± 4.77	10.81 ± 3.71	9.00	0.00	661.94 ± 33.46	462.35 ± 31.07	27.65	0.00	5.63 ± 1.43	2.47 ± 0.43	13.38	0.00
t	0.16	2.23			0.69	5.03			3.37	2.56		
*p*	0.87	0.03			0.49	0.00			0.74	0.01		

***Note:*** before = before dialysis but after catheterisation; after = 1 week after the dialysis,

* *p* > .05, Δp < .05.

### Indicators of renal function change

Both the experimental and the control groups quickly recovered renal function after treatment. The experimental group recovered more quickly than the control group in all related indicators (e.g. Cre, CysC and serum K+) except for Ure, and the intragroup differences were significant (Cre: *p* = .03; CysC: *p* = .01; and serum K^+^: *p* = .02) (Table-IV).

**Table-IV:** An intragroup comparison of time for restoring the renal function indicators (*X̅* ± S), n = 40.

Indicator	Ure (d)	Cre (d)*	CysC (d)*	Serum K^+(^d)*
Experimental	1.89 ± 0.81	2.15 ± 0.93	1.68 ± 0.67	2.84 ± 0.62
Control	1.86 ± 0.78	2.79 ± 0.59	2.07 ± 0.71	3.13 ± 0.45
t	0.17	3.75	2.53	2.39
*p*	0.86	0.03	0.01	0.02

* *p* < .05.

### Effective rate comparison

One week after treatment was completed, the experimental group obtained an effective rate of 100%, whereas the effective rate in the control group was 87.5%, with 35 cases being effectively treated. The intragroup difference in the effective rate was significant, with the experimental group being more effectively treated than the control group (*p* = 0.02) (Table-V).

**Table-V:** A comparison of the experimental and control groups’ effective rate (*X̅* ± S), n = 40.

Group	Excellent	Effective	Ineffective	Effective rate*
Experimental	33	7	0	100%
Control	26	9	5	87.5%
χ^2^				5.33
*p*				0.02

* *p* < .05.

## DISCUSSION

In recent years, advanced cervical cancer treatment regimens are inclined to be more cross-disciplined and integrated.^5^ A combined regimen of radiotherapy, chemotherapy, dialysis and surgical intervention helps reduce patients’ pain, prolonging their survival time and improving their quality of life.^6^-^8^ In treating advanced cervical cancer, the diagnosis and treatment of combined urologic diseases is sometimes conventional. However, other occasions are severe and demand emergency intervention to remove the causative factor.^9^ No matter what kind of therapy is chosen during cervical cancer treatment, be it surgery, post-surgical radiotherapy or chemotherapy, the ureter within therapeutic range is always impaired, though in varying degrees. The consequences of such impairment include hydronephrosis, abnormal renal function and obstructive renal failure.^10^,^11^

Some patients fail to show significantly improved renal function after a urethral catheter is placed to relieve the obstruction.^12^ Their severe symptoms of the internal environment disturbance such as azotaemia and hyperkalaemia are not corrected as soon as expected without significant changes in creatinine, urea nitrogen or serum potassium on the following day after the ureteral stent is placed.^13^ For such patients, haemodialysis should be performed to decelerate the rapidly collecting toxins within the body and help patients to handle the critical stage, according to our experience. In such cases, haemodialysis appears to have a confirmative effect, as patients can recover sooner. There are two types of haemodialysis: high-flux and low-flux.^14^ The quality of haemodialysis remains closely linked to the way they are implemented.^15^ Obstructive renal failure derived from cervical cancer presents both chronic renal functional compensation and characteristics of an acute attack. For postrenal obstructive renal disease, abnormal pressure within the renal pelvis causes a rapid inhibition of glomerular filtration function but does not change the glomeruli filtering membrane permeability. As a result, toxic substances within the human body quickly accumulate, especially those large and moderate molecular substances like β_2_ micro-globulin, PTH and cysteine protease inhibitor. They further affect the recovery of patients’ renal function. High-flux dialysis uses a high-flux membrane in haemodialysis.^16^ It has a significantly higher flux than the common one and can better eradicate the moderate and large molecular solutes. The present study verified that the β_2_ micro-globulin, PTH and cysteine protease inhibitor decreased more sharply in the experimental group applied with high-flux dialysis than in the control group. The intragroup differences are significant (*p* < .05), indicating high-flux dialysis appears to be more advantageous in eradicating the large and moderate molecular toxins among cervical cancer patients whose renal function is not significantly improved after obstructive renal failure catheterisation.

The study by Svara further implies^17^ that high-flux dialysis using biocompatible membrane can better protect patients’ remnant renal function, reduce the inflammatory response and improve the human body’s nutritional status. Compared with common dialysis, high-flux dialysis may greatly reduce morbidity among patients.^18^ Our study also suggests that the experimental group has significantly lower IL-6, CRP and TNF-a than the control group after dialysis is completed. A decrease in inflammatory factors promotes renal function restoration and prognosis. Also, Cre, CysC and serum K+ are restored more quickly in the experimental group than in the control group, and the intragroup differences are statistically significant.

Whether the haemodialysis can eliminate large and moderate molecular substances in the blood directly affects the therapeutic effect.^19^ This study demonstrates a 100% effective rate for the experimental group but an 87.5% for the control group. This difference is significant (*p* = .02). Moreover, patients in the experimental group recovered their renal function significantly more quickly than those in the control group (*p* < .05).

Cervical cancer-induced renal failure is essentially different from the functional failure of the kidney itself. For the former, once the obstruction is removed, most patients recover within a short period. For patients in our experimental group, their renal function improved within 3-4 days after completing dialysis. However, despite improved renal function, the indwelling stent should remain and be regularly replaced. Borboroglu et al.^20^ confirmed in their study of cervical cancer patients with renal dysfunction that indwelling stents should replace every 2 to 4 months later. As constant progress has been made in material science, the interval of an indwelling stent made of various materials has been extended from the original to four months to the present one year, though at a high cost. The stents may be chosen as per the patients’ specific conditions.

### Limitations of the study

The present study also has some limitations. First, dialysis is set to last one week. The time may be further shortened, and a more individualised haemodialysis plan may be drafted with renal function recovery as a research focus. By determining the decrease in inflammatory factors and large and moderate molecular toxins, it is hoped to reduce the treatment period and cost. Second, the samples are insufficient, so the sample size may be further expanded to achieve a large sample clinical analysis. Third, this study lacks a follow-up visit. Although the patients’ symptoms are improved through a combination of catheter-aided obstruction removal with dialysis, a large difference exists regarding the toxin eradicating effect in vivo of the two types of dialysis. We are also carrying outd additional studies to analyse the long-term effect of patients after receiving those two different types of dialysis, including the differences in long-term damage of obstructive renal failure-induced toxin build-up to other tissues and organs. Through the study, we will further elucidate the long-term benefits of high-flux dialysis to patients.

## CONCLUSION

Taken together, high-flux dialysis enables cervical cancer patients without significantly improved renal function after obstructive renal failure catheterisation to recover more quickly. Their internal inflammatory factors and moderate/large molecular toxins are more radically drained. The general efficiency of high-flux dialysis is higher. Therefore, for patients showing no significant improvement in renal function after being catheterised, high-flux dialysis should be administered in time to help patients handle the critical stage.

### Authors’ Contributions:

**CZ and DX** designed this study and prepared this manuscript and are responsible and accountable for the accuracy or integrity of the work.

**LA** collected and analysed clinical data.

**LZ** significantly revised this manuscript.
